# Prevalence, Identification and Antibiotic Resistance of *Gallibacterium anatis* Isolates from Chickens in Poland

**DOI:** 10.3390/pathogens12080992

**Published:** 2023-07-28

**Authors:** Olimpia Kursa, Grzegorz Tomczyk, Agata Sieczkowska, Anna Sawicka-Durkalec

**Affiliations:** Department of Poultry Diseases, National Veterinary Research Institute, 24-100 Puławy, Poland; gtomczyk@piwet.pulawy.pl (G.T.); agata.sieczkowska@piwet.pulawy.pl (A.S.); anna.sawicka@piwet.pulawy.pl (A.S.-D.)

**Keywords:** *Gallibacterium anatis*, prevalence, isolation, antibiotic resistance, chickens

## Abstract

The Gram-negative bacterium *Gallibacterium anatis* is part of the normal avian respiratory, intestinal and reproductive tract microflora and can be transmitted horizontally and vertically. With the coexistence of other relevant factors, *G. anatis* becomes an opportunistic pathogen, economically damaging to the poultry industry. This bacterium’s prevalence and molecular epidemiology were investigated, and the antimicrobial treatment options for *G. anatis* infection in chicken flocks in Poland were assessed. Tracheal samples from 182 flocks were collected between April 2022 and March 2023. The bacterial prevalence was determined by PCR targeting the *gyr*B gene and 16–23S rRNA. *Gallibacterium anatis* was identified by matrix-assisted laser desorption/ionisation–time-of-flight mass spectrometry (MALDI-TOF) after culturing and PCR amplification. Isolates’ susceptibility to 11 antimicrobials was assessed with a disc diffusion test. Isolates were also tested for *gyr*B, *Gtx*A and *flfA* virulence genes and *bla*_ROB_, *aph*A, *tet*B and *tet*H antibiotic resistance genes by PCR. Forty-one flocks (22.5%) were positive through PCR. Antibiotic resistance was most frequently observed against tilmicosin, tylosin, enrofloxacin, amoxicillin, tetracycline and doxycycline. Multiple resistance to at least eight antibiotics occurred in 20% of isolates and to at least four in 100%. The occurrence of *gyr*B was noted in 100%, *Gtx*A was detected in 89%, and *flf*A was found in 14% of positive samples. The *tet*B gene was present in 61.0% of positive samples, *tet*H was in 36.0%, *aph*A was in 16.7%, and *bla*_ROB_ was in 5.6%. Significant differences were found in *G. anatis* isolates related to the presence of the virulence genes GtxA and gyrB and the presence of resistance genes (*p* < 0.05) associated with resistance to tetracyclines, β-lactams and aminoglycosides. The continued rise in the resistance of *G. anatis* to a broadening range of antibiotics is a major problem for the poultry industry worldwide, as well as for public health. The findings of this study may expand the knowledge of the pathogenicity of *G. anatis* in poultry.

## 1. Introduction

*Gallibacterium anatis*, previously known as *Pasteurella anatis*, belongs to the phylum Proteobacteria, class Gammaproteobacteria and family *Pasteurellaceae*. It is a bacterium with pleomorphic cell morphology, and it is known as a commensal inhabitant of the respiratory, intestinal and reproductive tracts in poultry [1,2].

The pathogenicity of *G. anatis* depends on many factors related to the bacterial strain and the age, stress exposure and immune status of the bird [1,3,4]. In addition to these determinants, the severity of disease symptoms can be influenced by co-infection with other bacteria or viruses that damage the respiratory tract or cause immunosuppression [4,5]. Environmental factors can also exacerbate the disease. Mixed infection with *G. anatis* with viruses and bacteria such as *Escherichia coli*, *Avibacterium paragallinarum* and *Mycoplasma gallisepticum* may increase the rate of disease, resulting in increased morbidity and mortality [3,4,5,6]. The clinical form of the disease can lead to economic losses in the poultry industry by causing respiratory problems in birds and a wide range of pathological lesions in the ovaries and oviducts of laying hens. Infection of the reproductive tract of chickens can reduce egg production by 8–10%. In roosters, *G. anatis* is responsible for changes associated with reduced semen quality [4,5,7,8]. This opportunistic bacterium is responsible for the occurrence of many clinical signs, causing oophoritis, salpingitis, peritonitis and enteritis. Infections often remain undiagnosed [9].

Strains of *G. anatis* are widespread worldwide and have been isolated from various poultry species, such as chickens, turkeys, geese and ducks, but also partridges, guinea fowl and wild birds. It has been isolated in many countries in Europe, Asia and Africa, as well as the United States [5,10]. The widespread prevalence of *G. anatis* in poultry appears to be related to the route of its transmission in flocks. The main transmission route of *G. anatis* appears to be the respiratory system, but vertical transmission through the trans-ovarian, -oviduct and -eggshell routes is also possible, which was experimentally demonstrated in embryonated eggs. *Gallibacterium anatis* penetration into eggs is highly pathogenic to developing chicken embryos [9,10]

These bacteria can also instigate disease in non-avian species, in which they are involved in the pathogenesis of respiratory tract infections, such as tracheitis or aerosacculitis, and genital tract infections, such as salpingitis and oophoritis in cattle, horses, pigs, sheep and rabbits [1,9,11,12]. A recently described case of a woman who was in very bad health after renal transplantation was probably infected with *G. anatis* through food and developed bacteraemia and diarrhoea, which indicates that there are no species barriers to *G. anatis* [13].

Knowledge related to the virulence genes and antimicrobial susceptibility of *G. anatis* isolates in laying hens remains limited. The virulence factors of *G. anatis* involved in the colonisation and invasion of the tracheal epithelium, oropharyngeal tissues and oviducts, which include GtxA, bacterial fimbriae and tetracycline resistance determinants, are widespread and often found in multidrug-resistant bacterial species. Studies conducted around the world are revealing a large number of *G. anatis* strains showing multidrug resistance to three or more antibiotics. The growing multidrug resistance of *G. anatis* to antimicrobials is causing increased concern. Additionally, its range of host species being wide and not only avian makes it an important pathogen for its effect not only on livestock industries but also on public health. The purpose of this study was to present the prevalence of *G. anatis* in chicken flocks, as well as to assess the resistance of isolates to antibiotics. Additionally, we examined the presence of virulence and resistance genes for several classes of antibiotics.

## 2. Materials and Methods

### 2.1. Sample Collection

A total of 182 chicken flocks (162 layers and 20 broilers) from different parts of Poland were examined for *G. anatis* infection in 2022 and 2023. Tracheal swab samples were brought to the Department of Poultry Diseases at the National Veterinary Research Institute in Poland as part of a routine diagnostic test and monitoring programme. All examined birds were floor-reared, and most of them showed no respiratory or reproductive clinical signs. Some of the birds showed respiratory signs in the form of rales and coughing, and some of them had swollen heads.

### 2.2. DNA Extraction

Tracheal swabs were pooled separately into tubes containing Tris-EDTA buffer and processed for DNA extraction. Genomic DNA was extracted using a QIAamp DNA Mini kit (Qiagen, Hilden, Germany) according to the manufacturer’s recommendations. The quantity and quality of the DNA were determined using the NanoDrop 1000 system (Thermo Scientific, Waltham, MA, USA). DNA extraction from the Tris-EDTA used for sample preparation was conducted as a negative control. Samples were frozen at −20 °C until further analysis.

### 2.3. Real-Time PCR

To detect *G. anatis* by real-time PCR, primers complementary to the *gyr*B gene were used as described by Wang et al., 2016 [14], with slight modifications. The reaction was carried out using a QuantiTect Probe PCR Kit (Qiagen, Hilden, Germany) in a total volume of 25 μL with 1.3 μL of each 10 μM primer, 0.5 μL of probe, 7.4 μL of distilled water and 2 μL of DNA in an ABI 7500 thermal cycler (Applied Biosystems, part of Thermo Fisher Scientific, Norwalk, CA, USA) under the following conditions: 95 °C for 3 min and 40 cycles of 95 °C for 3 s. The fluorescence data were collected during a 60 °C for 32 s annealing–extension step.

### 2.4. PCR

PCR was conducted using previously described specific *G. anatis* primers that amplify the 16S–23S rRNA gene [15]. The PCR assays were performed on positive samples obtained in real-time PCR. The reaction mixture contained Taq PCR Master Mix (Eurx, Gdańsk, Poland) in a volume of 12.5 μL, 1.5 μL of each 10 μM primer and 7.5 μL of distilled water with the addition of 2 μL of DNA to give a total reaction volume of 25 μL. The PCR procedure included an initial incubation for 1 min at 95 °C, 35 cycles for 40 s each at 95 °C, annealing at 50 °C for 40 s, and extension at 72 °C for 40 s, with a final extension at 72 °C for 2 min. The PCR amplicons were separated by electrophoresis on a 2% agarose E-gel plate (Invitrogen, part of Thermo Fisher Scientific, Waltham, MA, USA) containing ethidium bromide and were visualised by ultraviolet transillumination. The method was used to identify *G. anatis* from swab samples as well as isolates.

### 2.5. Isolation and Identification of Gallibacterium anatis

The supernatant of the swabs (10 μL) from samples positive through PCR was inoculated onto Columbia agar plates with 5% sheep’s blood and incubated at 37 °C under a 5% CO_2_ atmosphere for 24 h. The *G. anatis* colonies were verified by matrix-assisted laser desorption ionisation–time-of-flight mass spectrometry (MALDI-TOF MS). The bacterial colonies from the agar plate were transferred to the MALDI target plate and mixed with formic acid and α-cyano-4-hydroxycinnamic acid matrix solution. All mass spectra were analysed with Bruker Daltonics software (Bruker Corporation, Billerica, MA, USA).

### 2.6. Antibiotic Resistance

The antimicrobial susceptibility of *G. anatis* was determined by using the disc diffusion method with 11 different drugs (Oxoid discs, Basingstoke, UK). The test applied a bacteria volume of 100 uL of 1.5 × 10^7^ CFU/mL (0.5 McFarland scale), distributed uniformly onto the Columbia agar with 5% sheep’s blood. Eleven antibiotics from nine classes were used: florfenicol (FFC, 30 μg)—quinolone class; doxycycline (DO, 30 μg) and tetracycline (TE, 30 μg)—tetracycline class; amoxicillin (AML, 25 μg)—β-lactam class; gentamicin (CN, 10 μg)—aminoglycoside class; enrofloxacin (ENR, 5 μg)—fluoroquinolone class; colistin (CT, 50 μg)—polymyxin class; ceftazidime (CAZ, 30 μg)—cephalosporin class; chloramphenicol (C, 30 μg)—chloramphenicol class; tilmicosin (TIL, 15 μg) and tylosin (TY, 30 μg)—macrolide class. The inhibition zones were interpreted visually. Eleven out of forty-one *Gallibacterium* isolates could not be recultivated after PCR identification; the DNA obtained from them was saved for further study.

### 2.7. Virulence and Resistance Genes

In addition, isolates of *G. anatis* were tested for the presence of the virulence genes *gyr*B, *Gtx*A and *flf*A. All samples were also tested for the presence of the antibiotic resistance genes *bla*_ROB_, *aph*A, *tet*B and *tet*H. A PCR method was used for both test steps with the starters described earlier [12] and DNA extracted from the *G. anatis* isolates using a QIAamp DNA Mini kit (Qiagen, Hilden, Germany) according to the manufacturer’s recommendations.

### 2.8. Statistical Analysis

Venn diagrams were constructed showing the number of shared virulence and resistance genes. Statistical analysis was carried out using one-way ANOVA and the Mann–Whitney test to determine the statistical significance of the presence of virulence and resistance genes. The value of *p* < 0.05 was considered statistically significant. Statistical analyses were performed using the Social Science Statistics program (www.socscistatistics.com 3 April 2023).

## 3. Results

### 3.1. Isolation and Identification

All the swab samples were tested by real-time PCR, and confirmed isolates of G. anatis (*n* = 41) were obtained from layer chickens. Twenty-two of them dated from 2023 and nineteen were from 2022. All samples positive in the real-time PCR were subjected to a specific PCR and had an amplicon of approximately 1030 bp. The prevalence of *G. anatis* in chicken flocks was 22.5%. Colony growth was obtained for 30 samples that were confirmed by MALDI-TOF MS. Other samples showed contamination related to overgrowth bacteria such as Proteus sp. All positive samples had a colony suggestive of the haemolytic biovar of *G. anatis*.

### 3.2. Antibiotic Resistance

In this study, 11 different antibiotics from different classes were used. The percentages of the 30 isolates with antibiotic resistance are shown in Table 1. The most frequently resisted antibiotics were enrofloxacin (100%), tilmicosin (100%), tylosin (100%), amoxicillin (83%) and tetracycline (80%). *Gallibacterium anatis* isolates showed less resistance to doxycycline (39%), chloramphenicol (33%), florfenicol (22%), gentamicin (18%), colistin (17%) and ceftazidime (11%). Resistance to nine and eight antibiotics occurred in 10% of isolates, and resistance to seven and six antibiotics was noted in 6.7% and 13.3%, respectively. The largest proportion of isolates were resistant to at least five antibiotics, at 43.3%, and the next largest to four antibiotics, at 16.7%.

### 3.3. Presence of Virulence and Resistance Genes

**Figure 1:**
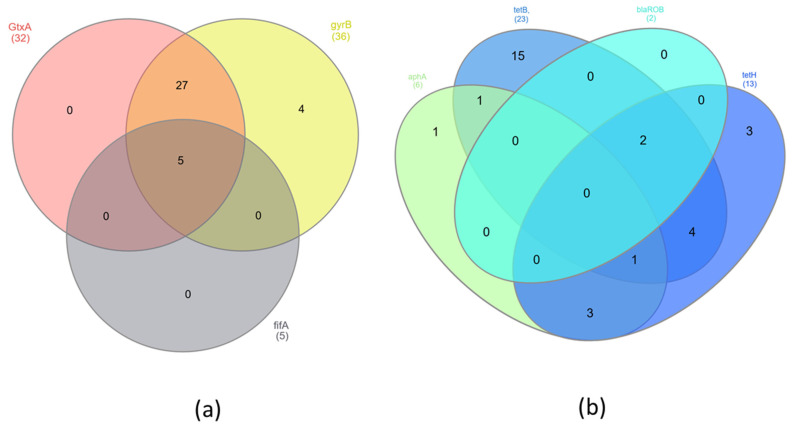
Venn diagrams showing presence of shared (**a**) virulence genes and (**b**) resistance genes in *G. anatis* isolates.

**Figure 2:**
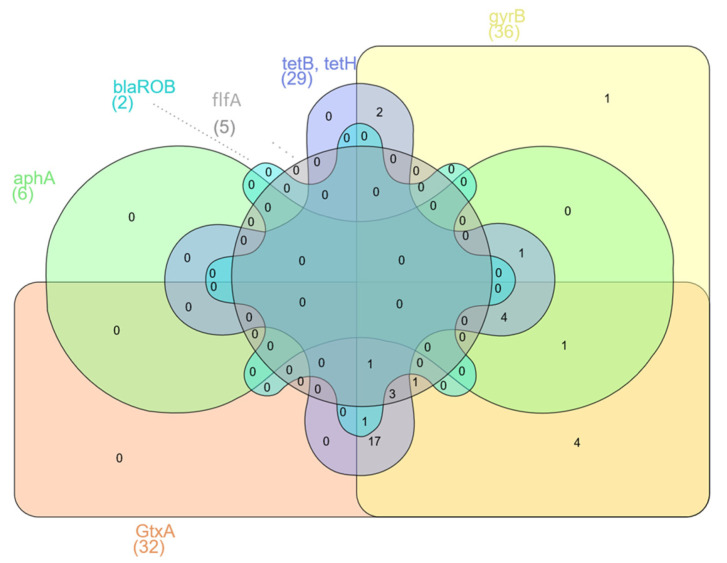
Venn diagram showing presence in the *G. anatis* isolates of shared resistance and virulence genes.

The presence of virulence and resistance genes was investigated in 36 samples by PCR. Five samples were excluded from the study because of poor DNA quality. The virulence gene *GtxA* was in 89% of isolates, *gyr*B was in 100%, and *flf*A was in 14% (Table 2). There were no statistically significant differences (*p* < 0.05) in the presence of *Gtx*A and *gyr*B genes. However, statistically significant differences (*p* < 0.05) were found between occurrences of the virulence genes *Gtx*A, *gyr*B and *flf*A. The antibiotic resistance gene *tet*B was found in 61.1% of isolates, *tet*H was in 36.1%, *aph*A was in 16.7%, and *bla*_ROB_ was in 5.6% (Table 2). The *Gtx*A and *gyr*B genes were present in 27 (75%) isolates. However, all three tested virulence genes were identified in only five (13.2%) isolates (Figure 1a). The largest group of resistance genes present in *G. anatis* isolates were the *tet*B and *tet*H genes. The presence of both of these genes was found in seven (19.4%) isolates; additionally, the *bla*_ROB_ gene was also detected in two of them, and the *aph*A gene was identified in one of them (Figure 1b). The largest number of isolates (*n* = 17) contained both virulence genes *Gtx*A and *gyr*B but also at least one of the resistance genes—*tet*B or *tet*H. Three isolates (8.3%) had all of the previously mentioned genes and additionally the *flf*A gene. In four (11.1%) isolates of *G. anatis*, besides two virulence genes (*Gtx*A and *gyr*B), the resistance genes *tet*B, *tet*H and *aph*A were found (Figure 2). Significant differences were found associated with the presence of the virulence genes *Gtx*A and *gyr*B and the presence of resistance genes (*p* < 0.05) associated with resistance to tetracyclines, β-lactams and aminoglycosides.

## 4. Discussion

*Gallibacterium anatis* has been one of the pathogens causing reproductive problems in poultry in recent years. It is a component of the normal microbiota of chickens, but under certain circumstances, it can be a problem for bird health. There are many factors influencing the development of *G. anatis* infection. Some are host-related factors, such as age, stress or hormones, and others are environmental factors, such as seasonal changes or cold stress [1,16,17]. The epidemiological status of infections depends heavily upon the pathogenicity of the strain, the route of infection and the presence of a secondary bacterial agent, such as *Mycoplasma gallisepticum*, *M. synoviae*, *Ornithobacterium rhinotracheale* or *E. coli* [5,9,18,19,20,21]. Viral agents can also facilitate the development of the clinical form of the associated disease [22]. The presence of *G. anatis* in chicken flocks has been reported in Europe (in Germany, Denmark, Belgium and Austria), but also outside Europe (in Turkey, Morocco, Egypt and the USA) [9,11,21,22].

Two *G. anatis* biovars, one haemolytic and the other non-haemolytic, can be found in the respiratory tract and the lower genital tract of birds. In poultry, the haemolytic *G. anatis* biovar has been associated with systemic infections, including pericarditis, perihepatitis, peritonitis and septicaemia [3,5,23]. In this study, the presence of *G. anatis* was detected in the respiratory system of laying hens, in which, in some flocks, a decrease in laying was noted, as well as respiratory problems and swollen heads in parts of the flocks. The identification of *G. anatis* in chicken flocks in Poland was confirmed by real-time PCR and PCR methods, and its prevalence was 22.5%. The isolated species, including its biovar, was *G. anatis* biovar haemolytica. The range of tissue tropism of this *G. anatis* species is very wide and can include the respiratory, gastrointestinal and reproductive systems.

Many papers in recent years have reported that *G. anatis* is increasingly resistant to antibiotics. The frequency of multidrug resistance to different classes of antibiotics is also rising [1,24,25], which is demonstrated in this work. The current study determined the susceptibility of *G. anatis* isolates to eleven antimicrobial agents from the nine classes. Resistance to at least four antibiotics was shown in 100% of the tested strains. The highest percentage of isolates was the percentage resistant to five antibiotics—43.3%. Resistance to at least eight antibiotics was shown by 20% of field isolates. The highest rates of resistance were to macrolide and fluoroquinolone classes of antibiotics, which has been confirmed in several other studies in recent years [9,11,26]. In this study, *G. anatis* strains showed the greatest resistance equally to tylosin, tilmicosin and enrofloxacin—100%—which is similar to the results of other studies in the case of tylosin [1,24,25]. In the case of enrofloxacin, the rate is much higher than was observed among isolates in the USA (0%), Germany (33.3%) or Austria (58.2%) [21,22,27]. Our data on the susceptibility of *G. anatis* showed that 22% of isolates were resistant to florfenicol, which belongs to the quinolone class; this proportion was higher than in other reports from the EU [21,27]. However, other studies showed similar results to those of the present research for the antibiotics of the tetracycline class: doxycycline and tetracycline. In our study, the resistance levels for these antibiotics were 39% and 80%, respectively. Resistance to an antibiotic from the β-lactam class (amoxicillin) was 83%, much higher than the resistance of isolates to this antibiotic recorded by other authors [3,12,21,27]. It should be underlined that one isolate of *G. anatis* resistant to eight classes of antimicrobials and three isolates resistant to seven classes of antimicrobials were identified. Thirty-three percent of the isolates were resistant to four different classes of antibiotics, and this represented the largest group of *G. anatis* isolates. The results of antibiotic resistance and multidrug resistance obtained from *G. anatis* field isolates are very alarming and indicate the need to reduce the use of antibiotics in poultry production.

Several resistance genes in *G. anatis* isolates were identified by PCR. Statistical differences (*p* < 0.05) in the occurrence of antibiotic resistance genes were observed. All *G. anatis* isolates possessed at least one of the resistance genes *tet*B, *tet*H, *aph*A or *bla*_ROB_. The tetracycline resistance gene *tet*B was the gene with the highest carriage rate, at 61%, and the rate for *tet*H was 36%, which is consistent with the results of Bojesen et al. (2011) and Abelazeem et al. (2022) [12,28]. Sixteen percent of *G. anatis* isolates had both the *tet*B and *tet*H genes. The detection of these genes in *G. anatis* isolates confirms their high antibiotic resistance to tetracycline and doxycycline. Aminoglycoside antibiotic resistance genes were investigated by attempting to detect the *aph*A resistance gene, which was identified and occurred in 17% of isolates in this study. Antibiotic resistance to gentamicin in the tested isolates was at similar levels. The presence of this gene was found to coincide with the *tet* genes in 14% of isolates. Statistical differences (*p* < 0.05) in the presence of antibiotic resistance genes *tet*B and *aph*A were observed. Among *G. anatis* isolates, low levels were found for the *bla*_ROB_ gene, which is a gene conferring resistance to antibiotics of the β-lactam class. Unfortunately, the low percentage of the gene detected does not coincide with the high antibiotic resistance of the isolates to amoxicillin. Samples in which the *bla*_ROB_ gene was found also contained *tet*B and *tet*H (Figure 1). The differences in the occurrence of these genes were statistically significant (*p* < 0.05). To expand knowledge in this area, future studies should be conducted on a higher number of resistance genes.

Additionally, we identified isolates containing virulence genes among *G. anatis*-positive chicken flocks. An 89% proportion of *G. anatis* isolates harboured the *Gtx*A gene, which enables the production of RTX-like cytotoxins and accounts for the haemolytic and leukotoxic activities of *G. anatis* (Figure 1) [29]. Studies have shown that the *Gtx*A protein has a significant inhibitory effect on lymphocyte growth. It is also considered an important pathogenic factor, affecting cell permeability and the expression of inflammatory factors, causing cell damage and apoptosis and significantly mediating the pathogenesis of salpingitis in poultry [29,30,31]. These results are comparable to data previously presented by other authors [30], which also relate to the presence of another virulence gene often used to identify *G. anatis* infections in chickens and turkeys. The gene *gyr*B encodes the ATPase domain of DNA gyrase, which is necessary for the replication of DNA in *G. anatis*, and it was detected in this study in all positive isolates. *Gallibacterium anatis* has the ability to adhere to glycoprotein receptors on the mucous membranes of birds. This is possible due to the presence of fimbriae, which have different sizes and shapes and are F17-like. Different types of fimbriae involved in the processes of colonisation and tissue adhesion were identified and grouped [10,12,32]. The *flf*A gene was another virulence gene detected in this study. The content of this gene in *G. anatis* isolates was much lower than those of *Gtx*A or *gyr*B, which is consistent with another study (Figure 1) [12].

*G. anatis* infections can lead to a wide range of serious clinical signs in many parts of the avian body. The bacterium can colonise many important organs in the host organism. In addition, the alarming increase in its multidrug resistance to an ever-increasing range of antibiotics noted around the world can lead to infections and treatment failures in poultry flocks.

## 5. Conclusions

In this study, we showed the prevalence of *G. anatis* in laying chickens in Poland. The findings from the current study show the multidrug resistance of the isolated strains and the presence of genes related to the virulence and resistance of *G. anatis* bacteria isolated from laying chickens. The results of this study may expand the knowledge of the pathogenicity of *G. anatis* in poultry.

## Figures and Tables

**Table 1:** Antimicrobial susceptibility of the 30 Gallibacterium anatis isolates.

Antibiotic	Dose (μg)	Resistant Isolates (%)
Enrofloxacin	5	100
Tilmicosin	15	100
Amoxicillin	25	83
Doxycycline	30	39
Ceftazidime	30	11
Tylosin	30	100
Gentamicin	10	18
Chloramphenicol	30	33
Colistin	50	17
Florfenicol	30	22
Tetracycline	30	80

**Table 2:** Presence of virulence and resistance genes in 36 G. anatis isolates.

Gene	Carriage in Isolates %
*Gtx*A	89.0
*flf*A	14.0
*gyr*B	100
*aph*A	16.7
*tet*B	61.1
*tet*H	36.1
*bla*_ROB_	5.6

## Data Availability

The data presented in this study are available on request from the corresponding author. The data are not publicly available because of legislation protecting privacy.
